# BMI and an Anthropometry-Based Estimate of Fat Mass Percentage Are Both Valid Discriminators of Cardiometabolic Risk: A Comparison with DXA and Bioimpedance

**DOI:** 10.1155/2013/862514

**Published:** 2013-12-24

**Authors:** Benno Krachler, Eszter Völgyi, Kai Savonen, Frances A. Tylavsky, Markku Alén, Sulin Cheng

**Affiliations:** ^1^Department of Health Sciences, University of Jyväskylä, P.O. BOX 35 (L), 40014 Jyväskylä, Finland; ^2^Kuopio Research Institute of Exercise Medicine, Haapaniementie 16, 70100 Kuopio, Finland; ^3^Department of Public Health and Clinical Medicine, Occupational and Environmental Medicine, Umeå University, 901 85 Umeå, Sweden; ^4^Department of Preventive Medicine, University of TN Health Science Center, Memphis, Tennessee 38163, USA; ^5^Department of Clinical Physiology and Nuclear Medicine, Kuopio University Hospital, 70211 Kuopio, Finland; ^6^Department of Medical Rehabilitation, Oulu University Hospital and Institute of Health Sciences, University of Oulu, 90029 Oulu, Finland; ^7^School of Kinesiology, Shanghai University of Sport, Shanghai 200438, China

## Abstract

*Objective*. To determine whether categories of obesity based on BMI and an anthropometry-based estimate of fat mass percentage (FM% equation) have similar discriminative ability for markers of cardiometabolic risk as measurements of FM% by dual-energy X-ray absorptiometry (DXA) or bioimpedance analysis (BIA). *Design and Methods*. A study of 40–79-year-old male (*n* = 205) and female (*n* = 388) Finns. Weight, height, blood pressure, triacylglycerols, HDL cholesterol, and fasting blood glucose were measured. Body composition was assessed by DXA and BIA and a FM%-equation. *Results*. For grade 1 hypertension, dyslipidaemia, and impaired fasting glucose >6.1 mmol/L, the categories of obesity as defined by BMI and the FM% equation had 1.9% to 3.7% (*P* < 0.01) higher discriminative power compared to DXA. For grade 2 hypertension the FM% equation discriminated 1.2% (*P* = 0.05) lower than DXA and 2.8% (*P* < 0.01) lower than BIA. Receiver operation characteristics confirmed BIA as best predictor of grade 2 hypertension and the FM% equation as best predictor of grade 1 hypertension. All other differences in area under curve were small (≤0.04) and 95% confidence intervals included 0. *Conclusions*. Both BMI and FM% equations may predict cardiometabolic risk with similar discriminative ability as FM% measured by DXA or BIA.

## 1. Introduction

Obesity is associated with cardiometabolic risk [1, 2]. The most commonly used definition of obesity is the body mass index (BMI). With the advance of more sophisticated measurements tools, assessment of body composition, rather than body mass, is increasingly used to study obesity-associated health risks [3, 4]. Although BMI correlates well with fat mass percentage (FM%) [5] it gives only a fair estimate of FM% [6], and individuals with large muscle mass may be misclassified as overweight or obese [7].

Dual-energy X-ray absorptiometry (DXA) devices estimate FM% with acceptable accuracy and have become the reference method for estimating body composition [8]. However, their drawbacks are radiation exposure, relatively high cost, and limited accessibility. Compared to DXA, bioimpedance analysis (BIA) has been shown to provide a good degree of accuracy in various populations of healthy subjects with stable hydration levels and within the normal range of body fat [9–11]. Bioimpedance is dependent not only on body composition (water content) but also on body size (cross-sectional areas in trunk and limbs). Since the proportions of body segments depend not only on weight, age, and gender but also on race, estimation equations for FM% need to be population specific. However, computational algorithms used to estimate FM% in commercial BIA units are generally proprietary and confidential and may be changed without notice. Further, population specific studies to validate these equations are either lacking or unpublished.

Equations that estimate FM% based on simple anthropometric measures such as weight, height, and waist circumference (FM% equations) overcome some of the shortcomings of DXA and BIA: they are simple and inexpensive and can be applied to existing epidemiological data. Most of the FM% equations have been validated against DXA measurements in different populations [12, 13]. Consequently, they have a lower degree of accuracy in estimating FM% than DXA measurements.

The hypothesis tested in this study is that this loss of accuracy in estimating FM% is irrelevant in the assessment of obesity-related cardiometabolic risk on a group level, and hence using weight and height-based FM% equations to categorize obesity yields a similar discriminative ability as DXA or BIA measurements. Further, we investigate whether any of the FM%-based measures of obesity improves on BMI. Thus, we intend to test the null-hypothesis that all these methods have similar predictive power for hypertension, impaired fasting glucose, dyslipidaemia, and the metabolic syndrome. If confirmed, FM% equations and/or BMI can be used to identify individuals with elevated cardiometabolic risk instead of DEXA and/or BIA. This offers considerable economic savings, both in clinical health care and research.

## 2. Materials and Methods

### 2.1. Study Population

The study population consisted of 40–79-year-old male (*n* = 205) and female (*n* = 388) healthy volunteers who resided in Central Finland and participated in a family study. Background information, including the health status, was collected via a self-administrated questionnaire. Written informed consent was obtained before the laboratory examinations. The Ethical Committee of the Central Finland Health Care District approved the study (Registration number: K-S shp:n dnro22.8.2008). All data were handled confidentially.

### 2.2. Cardiometabolic Risk Factors

#### 2.2.1. Measurements

Blood samples were taken in the morning between 0730 and 0900 after the subjects had fasted for 12 hours. Blood pressure (BP) was measured by the manual oscillometric method after 5 min rest. If women were in the pre menopausal state, the blood sample was drawn on the 5th day from the start of menstruation. Serum was separated within 30 minutes and stored at −80°C until analysis. Serum glucose, total and high-density lipoprotein (HDL) cholesterol and triacylglycerol concentrations were measured by enzymatic photometry on a Kone Pro Clinical Chemistry Analyzer (Thermo Clinical Labsystems Oy, Vantaa, Finland) with commercial kits. Low-density lipoprotein (LDL) cholesterol was calculated using the Friedewald equation [18].

#### 2.2.2. Definitions

In grading hypertension, we followed the definitions of the European Society of Hypertension [19] such as: (Grade 1 = systolic BP ≥ 140 and/or diastolic BP ≥ 90, Grade 2 = systolic BP ≥ 160 and/or diastolic BP ≥ 100, Grade 3 = systolic bp ≥ 180 and/or diastolic BP ≥ 110).

Dyslipidaemia is defined as either triacylglycerol ≥ 1.7 mmol/L or HDL cholesterol < 1.0 mmol/L men/1.30 mmol/L women. For Impaired fasting glucose, we examined both the stricter International Diabetes Federation (IDF) sponsored 5.6 mmol/L (100 mg/dL) and higher level of 6.1 mmol/L (110 mg/dL) recommended by the WHO [20].

For definition of metabolic syndrome, we adopted cutoffs values suggested by the common task force from the IDF and the American Heart Association/National Heart, Lung and Blood Institute (AHA/NHBLI) [17], requiring presence of at least three out of the following: (1) fasting plasma glucose ≥ 5.6 mmol/L (100 mg/dL); (2) triacylglycerol ≥ 1.7 mmol/L (150 mg/dL); (3) HDL Cholesterol < 1.0 mmol/L men/1.30 mmol/L women (40 and 50 mg/dL, resp.); (4) waist circumference > 102 cm men/88 cm women; and (5) systolic BP ≥ 130 mm Hg OR diastolic BP ≥ 85 mm Hg.

### 2.3. Anthropometric Measurements

All measurements were performed after an overnight fast. Participants were weighed without shoes and with light clothes. Height was determined to the nearest 0.1 cm using a fixed wall-scale measuring device. Weight was determined within 0.1 kg for each subject using an electronic scale, calibrated before each measurement session. BMI was calculated as weight (kg) per height (m^2^). Waist circumference was measured with a measuring tape at the largest circumference location between landmarks of the most proximal iliac and the most distal rib bone as a mean value of two measurements.

### 2.4. Anthropometry-Based FM% Estimation Equations (FM% Equations)

From a literature search we found five different equations for FM% estimation in adult men and women (Table 1). Four equations are based on age, gender, weight, and height [12–14, 16] and one utilized in addition waist circumference [15]. A combination of prediction equations has been shown to provide a better estimate than relying on one equation only [21]. The relationship between anthropometric measures and FM% is different, not only among the main racial categories [6, 15] but, even between different countries within Europe [22]. The population of Finland is one of the European Union (EU) genetically most distinct populations [23]. Therefore, instead of using all 5 equations we eliminated the 2 with the largest bias compared to DXA. Thus the chosen equation relevant for a Finnish population is the arithmetic mean of the three FM% equations with the lowest bias compared to DXA:

(1a) $$\begin{array}{c} \text{FM}\%=\frac{-24.18+1.181*\left(\text{weight}/\text{height}\right)}{\text{weight}}\;\text{for}\;\text{women}, \end{array}$$

(1) FM%  =−30.84+1.120∗(weight/height)weight  for  men.

For the first equation see [18]

(2) FM%=64.5−848∗(1BMI)+0.079∗age−16.4∗sex−0.05∗sex∗age+39.0∗sex∗(1BMI).

For the second equation see [15]

(3) FM%=1.2∗BMI+0.23∗age−10.8∗sex−5.4.

For the third equation see [12] where sex= 1 for men and 0 for women.

### 2.5. DXA Measurements

Prodigy with software version 9.3 GE Lunar, Madison, WI, USA was used to estimate FM and FM%. Precision of the repeated measurements expressed as the coefficient of variation was 2.2% for FM.

### 2.6. Bioimpedance Measurements

InBody (720) (Biospace, Seoul, Korea) is a multifrequency impedance body composition analyzer. Total body water (TBW) was estimated with the manufacturer-provided device specific software from area, volume, length, impedance, and a constant proportion (specific resistivity). Fat free mass (FFM) was estimated by dividing TBW by 0.73. Readings of FFM and FM% are reported in this paper. Precision of the repeated measurements expressed as coefficient of variation was, on average, 0.6% for FM%.

### 2.7. Statistical Analysis

The concepts of reclassification index and integrated discrimination improvement were introduced by Pencina et al. [24] and described and illustrated by Cook and Ridker [25]. Briefly, the net reclassification index (NRI) compares two models that divide participants into different categories of risk for a dichotomous outcome. It is calculated as the net increase versus decrease in risk categories among case patients minus that among noncase participants:

(4) NRI=[Pr(up∖cases)−Pr(down∖cases)]−[Pr(up∖non-cases)−Pr(down∖non-cases)].

Thus, a positive NRI means that the comparison method has a better predictive power than the reference method.

The integrated discrimination improvement (IDI) describes the mean difference in predicted probabilities between case patients and noncase participants for two models. It is calculated from individual predicted probabilities for each participant in the respective models:

(5) IDI=(ave  pcases−ave  pcontrols)comparison  method  −  (ave  pcases−ave  pcontrols)reference  method,

where *p* is the predicted probability for each participant. Predicted individual probabilities are derived from gender-specific logistic regressions with obesity category as independent variable, 10-year age groups as covariates, and the cardiometabolic risk factor in question as outcome. The difference in slopes is a measure of improvement in the model. Thus, a positive IDI means that the comparison method has a better predictive power than the reference method.

Categories of obesity according to each specific obesity-estimation method were formed based on age and gender. Each of the 4 subgroups (men and women aged above and below 60 years, resp.) was sorted by degree of obesity, separately for each definition (i.e., BMI and various FM% measurements and estimates). Percentiles at BMI 25 and 30 were used to obtain corresponding cutoffs for obesity categories according to each of the FM%-measurement and estimation methods. Thus we obtained categories of obesity with identical numbers of subjects but partly different individuals sorted by degree of obesity according to the respective method. Due to small numbers of underweight and severely obese subjects we settled on the following categories: (1) normal BMI ≤ 24.9; (2) overweight BMI 25–29.9, and (3) obese BMI ≥ 30 (Table 2). Based on percentiles corresponding to these cut-offs, we categorized participants as normal, overweight, or obese according to each of the different FM%-estimates (DXA, BIA, FM%-prediction equations). Finally, we compared the predictive power of the categories of obesity with cardiometabolic risk factors (hypertension, impaired fasting glucose, dyslipidaemia, and metabolic syndrome) as outcomes. We used DXA-based categories of obesity as the reference for all other models. Furthermore, we made additional comparisons between obesity categories based on BIA and BMI.

**Figure 1:**
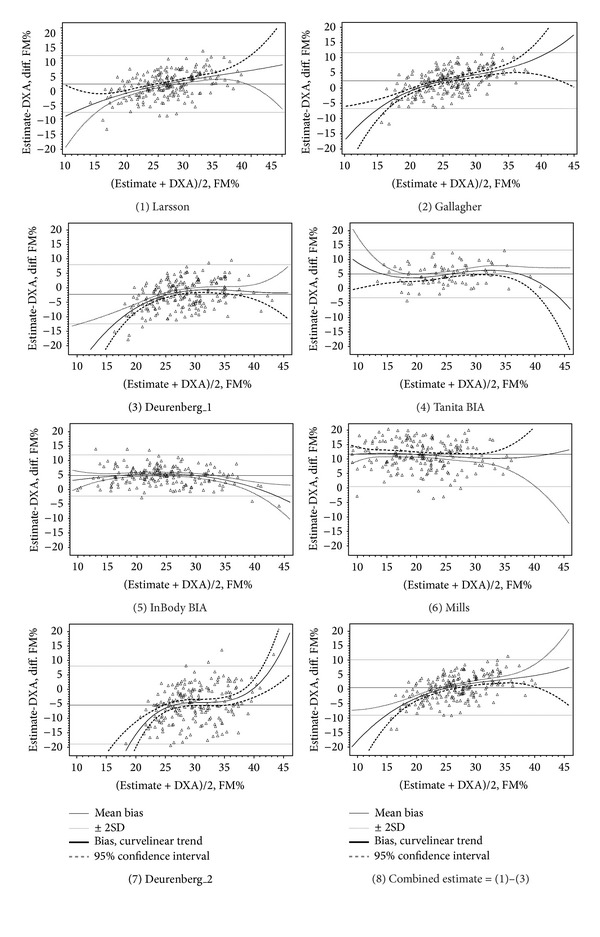
Bland-Altman plots for estimates of fat mass percent (FM%): DXA versus FM%-prediction equations and bioimpedance in men.

**Figure 2:**
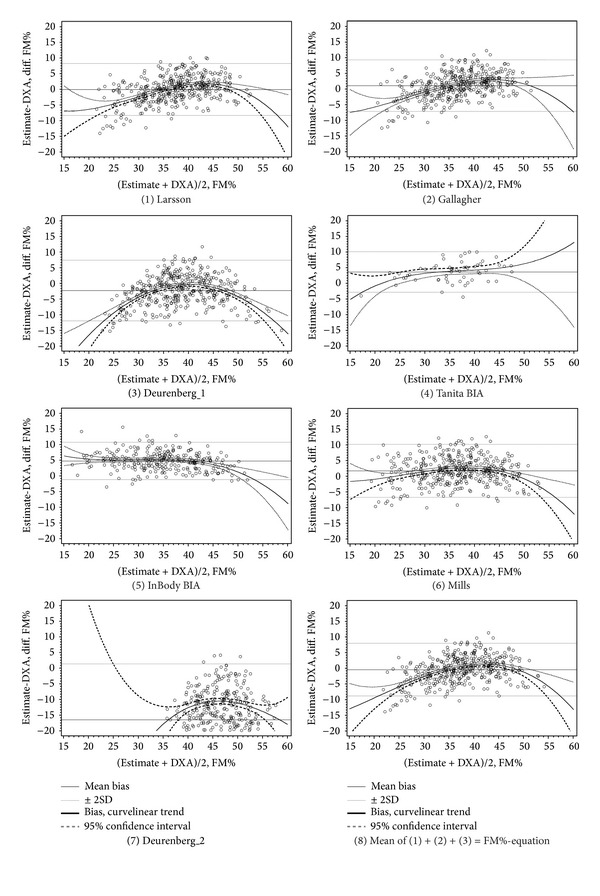
Bland-Altman plots for estimates of fat mass percent (FM%): DXA versus FM%-prediction equations and bioimpedance in women.

Bland-Altman plots were used to compare the mean difference of the various FM% equations to DXA (Figures 1 and 2). A comparison of the two BIA devices against DXA in our study population has been published previously [26].

For models using BMI and FM% as a continuous variables, nonnormally distributed variables were power-transformed. Distributions of the resulting variables fulfilled criteria for goodness of fit with normal distribution (Kolmogorov-Smirnov *P* > 0.15 and Anderson-Darling *P* > 0.25). However, results obtained from calculations based on transformed variables were identical with those obtained by nontransformed variables. This indicates that the ROCmodels are more robust with regards to the normal distribution assumption than suggested by Goddard and Hinberg [27].

Receiver operating characteristics were calculated from sex-specific logistic regressions with method specific obesity-categories and 10-year age categories as independent variables. The SAS-procedure: PROC LOGISTIC/roc-contrast was used to estimate differences in area under curve (AUC).

The Statistical Analysis System (SAS for Windows, version 9.2, SAS Institute, Carry, NC, USA) was used for all statistical evaluations. For calculation of net reclassification index and integrated discrimination improvement we adapted SAS-macros provided by Cook and Ridker as a supplement [25].

## 3. Results

Anthropometric and metabolic characteristics of the study population are given in Table 3. The mean values showed slight overweight and mild hypertension both in males and females. Fasting glucose was in the range of impaired fasting glucose in men but not in women (according to the lower cutoff of 5.6 mmol/L recommended by the IDF [28]).

Comparisons of predictive powers of the various obesity measures for hypertension grades 1 and 2 [19] and dyslipidaemia are given in Table 4. There were too few subjects with grade 3 hypertension to build stable mathematical models. BMI, FM%-equation- and BIA-based categories of obesity improved discrimination for grade 1 hypertension compared to DXA by 1.5–1.9%. BMI and FM% equation-based categories of obesity had 1.2% lower discrimination for grade 2 hypertension compared to DXA and 2.5–2.8% lower compared to BIA. BMI categories provided 2.5–3.7% improved discrimination regarding dyslipidaemia compared to both DXA and BIA. AUC, based on continuous variables, showed better prediction of grade 1 hypertension for BIA and FM% equations compared to DXA in women, but not in men. For prediction of grade 2 hypertension BIA was superior to DXA, BMI, and FM% equations. Differences in net reclassification did not reach significance level.

A comparison of the various obesity-measures ability to indicate different levels of impaired fasting glucose and metabolic syndrome is given in Table 5. Prediction of impaired fasting glucose with the lower cutoff was similar for all methods. Both BIA- and FM% equation-based categories outperformed DXA by 2.6% to 3.5% in predicting impaired fasting glucose of 6.1 mmol/L (110 mg/dL) or more. All methods had similar discrimination with regard to the metabolic syndrome. In the sex-specific ROC analyses, there were no significant differences in AUC. Again, differences in net reclassification did not reach significance level.

**Figure 3:**
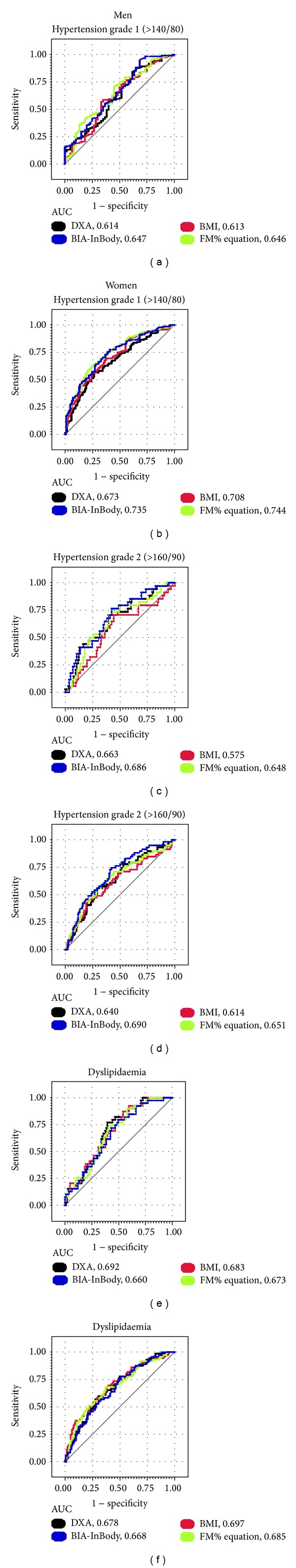
Receiver operating characteristic of DXA, BIA, BMI, and anthropometry-based estimate of fat mass percent (FM%-equation) as predictors of hypertension and dyslipidaemia. (a) In direct comparisons, the area under curve (AUC) for the anthropometry-based estimate of fat mass percentage (FM%-equation) is larger than AUC for BMI (*P* = 0.021). (b) AUC for the BIA InBody is larger than for DXA (*P* < 0.001). AUC for FM%-equation is larger than for both DXA and BMI (*P* < 0.001). (c) AUC for BIA InBody is larger than for BMI (*P* = 0.006) as is AUC for the FM%-equation (*P* < 0.001). (d) AUC for the BIA InBody is larger than for DXA and BMI (*P* < 0.001) and also larger than for the FM% equation (*P* < 0.044). AUC for the FM% equation is larger than for BMI (*P* < 0.001). (e) AUC for the FM%-equation is larger than for BIA InBody (*P* = 0.013). (f) There are no significant differences in areas under curve (AUC) for the different methods.

**Figure 4:**
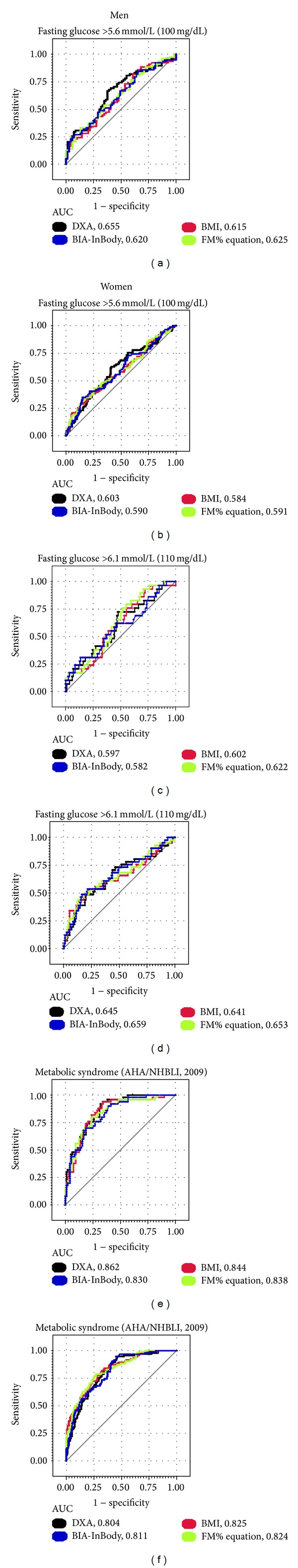
Receiver operating characteristic of DXA, BIA, BMI, and anthropometry-based estimate of fat mass percent (FM%-equation) as predictors of elevated fasting glucose and the metabolic syndrome. (a)–(f) There are no significant differences in direct comparisons of areas under curve (AUC) for the different methods.

The receiver operated characteristics of the different obesity measures as predictors of cardiometabolic risk factors are shown in Figures 3 and 4. BIA-based categories were the best predictors of hypertension in men. In women, FM% equations were the best predictor of grade 1 hypertension and BIA-based categories were best predictors of grade 2 hypertension. For all other outcomes differences in AUC were small (<0.05).

**Figure 5:**
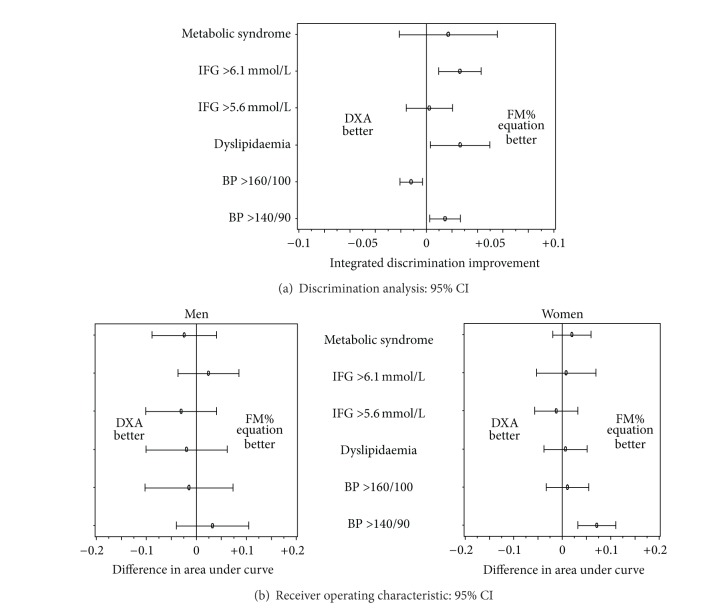
Comparison of DXA and anthropometry-based estimate of fat mass percent (FM% equation) as predictors of cardiometabolic risk factors. (a) Comparison of the integrated discrimination (= mean individual prediction of cases−mean individual prediction of referents) between categories of obesity based on DXA measurements and categories-based on anthropometry-based estimate of fat mass percentage (FM% equation) basis: whole study population, both men and women. (b) Difference in area under curve between fat mass % as a continuous variable measured by DXA and estimated by FM%-equation.

A comparison of 95% confidence intervals for differences in integrated discrimination and AUC between DXA and the anthropometry-based estimate is given in Figure 5. Integrated discrimination improvements varied between −3% and +6%. Differences in AUC were between −0.1 and +0.1 in men and between −0.5 and 0.1 in women.

## 4. Discussion

In this population of healthy, middle aged, and elderly Finns we found that anthropometry-based FM%-predictions and BMI had similar predictive power for obesity-associated cardiometabolic risk markers as FM% derived from DXA- or BIA measurements. Some of the studied obesity measures have small advantages in discriminating one single cardiometabolic risk factor. With regard to metabolic syndrome—which combines all of the studied risk factors—discriminative ability of all obesity measures is similar.

Our results are consistent with findings from other healthy populations that used DXA as reference method. In a multi ethnic survey of adults [5] BMI and waist circumference were comparable to DXA measurements of fat mass and FM% as assessed by their correlations with blood pressure, lipids, fasting glucose, C-reactive protein, and fasting insulin. In addition, BMI and waist circumference demonstrated similar abilities to distinguish between participants with and without metabolic syndrome. In a smaller study of elderly women [29] BMI and DXA-measured FM% had similar correlations with and discriminative ability of hypertension, dyslipidaemia, and hyperglycaemia. In a study of Caucasian children [30], measures of total and central fat mass from DXA did not show an improved ability over BMI, to identify children with elevated systolic BP. A recent review of studies in patients with pulmonary disease [31] found no evidence that body composition calculated from BIA and anthropometry was better at predicting clinical outcomes than body composition calculated by simple anthropometry alone.

Conversely, a study combining weight-loss outpatients and hospital staff [32] found elevated concentrations of cardiometabolic risk factors in nonobese individuals according to BMI but obese based on FM%-categories. The reference method was air displacement plethysmography and—unlike our results—BMI was found to systematically underestimate the degree of obesity. However, this study utilized a different reference method, had identical FM% cutoffs for all age groups (18 to 80 years of age), and was conducted in a different ethnic group and a higher proportion of obese subjects. A higher prevalence of diseased subjects could be another potential explanation for the different outcome as measurements of body composition have been reported to have better discriminative and prognostic ability compared to BMI both during and after chronic disease [33–35].

Ethnicity influenced the methodology of our study. The relationship between anthropometric measures and FM% is different, not only among the main racial categories [6, 15] but even between different countries within Europe [22]. The population of Finland is one of the European Union's (EU) genetically most distinct populations [23] and with 96% of the residents born within the country [36] also one of EU:s most homogenous. This uniqueness was further confirmed by a European multi centre study developing BMI-based FM%-predictions based on BIA measurements in which the bias in the Finnish population differed from that of all other sites [37]. Since there are no official FM% cutoffs to categorize obesity and the most frequently used values are derived from BMI cutoffs in a US population [15, 38, 39], we settled for comparing percentile-based categories. Further, instead of using a mean of all available FM% equations, we selected the three that are most relevant for a Finnish population, based on bias versus DXA-values for FM%. As the two different anthropometry-based measures of adiposity performed equally well against DXA and BIA, selection of FM% equations is unlikely to have distorted our results.

Absence of data regarding prescribed medication is a further limitation of our study. Both antihypertensive and cholesterol-lowering agents weaken the association between obesity and hypertension/dyslipidaemia, as they attenuate the outcome (hypertension, dyslipidaemia) without changing the exposure we study (obesity). Neither DXA nor BIA measurements are part of routine health care in Finland. However, anthropometric measures are taken frequently in primary care and—if indicating obesity—often lead to further laboratory testing which may ultimately result in the prescription of lipid-lowering or antihypertensive drugs. Thus, in our comparison with DXA and BIA disregarding medication is likely to have resulted in underestimation of BMI and FM% equations as risk indicator.

Both reclassification index and integrated discrimination improvement are comparatively new statistical tools, which may complicate the interpretation of results. The reclassification index is exclusively based on participants that change risk categories between models. It provides no confidence intervals and, as in our study, it is difficult to judge if nonsignificant results imply small differences or lack of power. The integrated discrimination improvement takes into account all changes in individual probabilities between models and is, thus, a more sensitive measure. Receiver operated characteristics show a similar picture of only small differences in predictive power between FM%-measurements and anthropometry-based estimates of FM%. Thus, all three different statistical methods yielded similar results.

Measurement and estimation methods that are feasible in an epidemiological context may still have too wide a range of error to be used to predict individual risk levels in a clinical setting to predict individual risk levels. Although the mean bias of some of the weight and height based FM% estimates was smaller than that of bioimpedance, the standard deviations were consistently larger. Whether that lack of precision in estimating individual FM% translates into unacceptably imprecise estimates of individual cardiometabolic risks cannot be answered in the current study. Both reclassification index and integrated discrimination are used on the group level only and provide no measures of individual variability.

## 5. Conclusions

Our results suggest that for predicting cardiometabolic risk in healthy middle aged and elderly Finns at population level, anthropometry-based categories of obesity are equivalent to obesity categories derived from DXA and BIA measurements. Further, our results indicate that discriminative ability of anthropometry-based FM% equations and BMI are similar.

Low-cost measures of obesity can be utilized in screening for obesity related risk.

## Figures and Tables

**Table 1:** Anthropometry- and bioimpedance-analysis- (BIA-) based estimates of fat mass percentage (FM%) and their respective bias versus DXA measurements.

Predictor	Equation for estimating FM%	Men			Women			Combined	
		*n*	Mean bias^a^	SD	*n*	Mean bias^a^	SD	*n*	Mean bias^a^
(*1*) Larsson et al. [14]	Women: FM% = (−24.18 + 1.181 ∗ weight/height)/weight Men: FM% = (−30.84 + 1.120 ∗ weight/height)/weight	205	−1.3	4.7	388	0.1	4.1	593	−0.6
(*2*) Gallagher et al. [15]	FM% = 64.5 – 848 ∗ (1/BMI) + 0.079 ∗ age −16.4 ∗ sex^b^ − 0.05 ∗ sex^b^∗ age + 39.0 ∗ sex^b^∗ (1/BMI)	205	−2.4	4.6	388	−1.0	4.1	593	−1.7
(*3*) Deurenberg et al. [12]	FM% = 1.2∗BMI + 0.23∗age −10.8∗sex^b^ − 5.4	205	1.7	4.9	388	2.3	4.8	593	2.0
(*4*) Tanita BC 418 MA	bioimpedance-based proprietary algorithm	82	−4.8	3.9	58	−3.6	3.2	140	−4.2
(*5*) InBody (720)	bioimpedance-based proprietary algorithm	181	−4.6	3.4	273	−4.7	3.0	454	−4.6
(*6*) Mills [13]	Women: FM% = (−2.28 + 1.268 (weight/height) + 0.058 ∗ age)/weight Men: FM% = (−7.99 + 1.286 (weight/height) + 0.018 ∗ age)/weight	205	−11.7	5.3	388	−1.6	4.2	593	−6.7
(*7*) Deurenberg et al. [16]	FM% = −11.4 ∗ sex^b^ + 0.2 ∗ age + 1.294 ∗ BMI − 8	205	5.0	6.4	388	16.5	8.9	593	10.8
(*8*) FM%-equation	Arithmetic mean of equations (*1*)–(*3*)	205	−0.7	4.6	388	0.5	4.2	593	−0.1

^a^fat mass percentage.

^b^male: 1, female: 0.

**Table 2:** Anthropometric and metabolic characteristics of the study population.

	Men			Women		
	*n*	Mean	95% CI	*n*	Mean	95% CI
Age (years)	205	57	(55–58)	388	56	(55–57)
Height (cm)	205	176	(175–177)	388	163	(162–164)
Weight (kg)	205	82.5	(81.0–83.9)	388	70.6	(69.3–71.9)
BMI (kg/m^2^)	205	26.6	(26.2–27.1)	388	26.6	(26.1–27.1)
Fat mass (kg)	205	22.7	(22–23.6)	388	26.9	(25.9–27.9)
Fat mass (%)	205	27.1	(26–27.9)	388	37.1	(36.4–37.9)
Waist circumference (cm)	200	95	(93–96)	376	86	(85–87)
Chest circumference (cm)	166	102	(101–103)	269	98	(96–99)
Systolic blood pressure (mmHg)	201	146	(144–149)	377	142	(140–145)
Diastolic blood pressure (mmHg)	201	85	(84–87)	377	83	(82–84)
Fasting glucose (mmol/L)	166	5.8	(5.6–5.9)	301	5.6	(5.5–5.7)
Fasting insulin (*μ*IU/ml)	166	10.1	(6.5–14)	302	8.2	(7.5–9.0)
Total cholesterol (mmol/L)	167	5.3	(5.1–5.4)	302	5.5	(5.4–5.6)
HDL (mmol/L)	167	1.5	(1.4–1.6)	302	1.8	(1.7–1.9)
LDL (mmol/L)	167	3.1	(3–3.3)	302	3.1	(3.0–3.2)
Triglycerides (mmol/L)	167	1.5	(1.3–1.6)	302	1.2	(1.2-1.3)
Free fatty acids (mmol/L)	167	496	(450–542)	302	541	(502–580)

**Table 3:** Values in fat mass percentage (FM%) for the method-specific percentile corresponding to BMI percentiles at BMI 25 and 30, respectively.

	FM% cutoffs corresponding to BMI 25				FM% cut-offs corresponding to BMI 30			
	Men		Women		Men		Women	
Method^a^/age-group	<60	≥60 y	<60	≥60 y	<60	≥60 y	<60	≥60 y
DXA	24.0	26.1	36.7	34.7	32.3	37.5	44.0	43.8
BIA InBody^b^	19.3	23.0	31.5	31.9	28.7	34.0	38.7	40.8
FM%-equation^c^	24.0	26.3	35.3	37.4	30.1	31.7	41.6	43.8

^a^Method of measurement, based on which participants are classified in categories of obesity.

^b^Estimation of FM% with bioimpedance device InBody (720) (Biospace, Korea).

^c^Anthropometry-based estimation of FM%; arithmetic mean of FM% estimates according to prediction methods Deurenberg et al. [12], Gallagher et al. [15], and Larsson et al. [14].

**Table 4:** Prediction/discrimination of hypertension with degree of obesity as defined by dual-energy X-ray absorptiometry (DXA) bioimpedance analysis (BIA), an anthropometry-based estimate of fat mass percentage (FM% equation) and BMI.

								ROC analyses^n^					
Reference method/model^a^	New method/model^b^	*n* ^*c*^		Reclassification index, %^f^				IDI, %^k^		Men		Women	
		Cases^d^	Non-cases^e^	Net^g^	*P*_nri_^h^	Cases^i^	Non-cases^j^	*I*_integr._^l^	*P*_idi_^m^	ΔAUC^o^	*P* ^p^	ΔAUC	*P*
Hypertension^q^, grade 1 (≥140/90 mmHg)													
DXA	BIA InBody^r^	269	185	5%	0.214	−1%	6%	1.7%	0.017	0.03	0.127	0.06	0.000
	BMI	335	258	6%	0.220	2%	3%	1.9%	0.006	0.00	0.977	0.04	0.073
	Estimate^s^	335	258	6%	0.208	2%	3%	1.5%	0.019	0.03	0.383	0.07	0.000
BIA InBody	BMI	269	185	4%	0.360	1%	3%	0.5%	0.534	−0.03	0.330	−0.03	0.147
	Estimate	269	185	3%	0.502	0%	3%	0.1%	0.885	0.00	0.979	0.01	0.606
BMI	Estimate	335	258	0%	0.803	0%	0%	−0.4%	0.144	0.03	0.021	0.04	0.000

Hypertension, grade 2 (≥160/100 mmHg)													
DXA	BIA InBody	93	361	−1%	0.848	−4%	3%	1.4%	0.063	0.02	0.396	0.05	0.000
	BMI	117	476	−9%	0.128	−8%	−1%	−1.2%	0.049	−0.09	0.064	−0.03	0.255
	Estimate	117	476	−8%	0.154	−7%	−1%	−1.2%	0.036	−0.01	0.746	0.01	0.626
BIA InBody	BMI	93	361	−9%	0.161	−8%	−1%	−2.5%	0.003	−0.11	0.006	−0.08	0.000
	Estimate	93	361	−10%	0.096	−9%	−1%	−2.8%	0.001	−0.04	0.309	−0.04	0.044
BMI	Estimate	117	476	1%	0.682	1%	0%	0.0%	0.870	0.07	0.001	0.04	0.000

Dyslipidaemia^t^													
DXA	BIA InBody	111	304	−2%	0.616	−5%	3%	−0.1%	0.928	−0.03	0.161	−0.01	0.510
	BMI	124	345	6%	0.320	2%	4%	3.5%	0.015	−0.01	0.816	0.02	0.378
	Estimate	124	345	4%	0.496	0%	4%	2.7%	0.040	−0.02	0.640	0.01	0.766
BIA InBody	BMI	111	304	8%	0.149	6%	2%	3.1%	0.022	0.02	0.568	0.03	0.162
	Estimate	111	304	6%	0.240	5%	2%	2.5%	0.044	0.01	0.734	0.02	0.390
BMI	Estimate	124	345	−2%	0.237	−2%	−1%	−0.8%	0.111	−0.01	0.598	−0.01	0.148

^a^Method of measurement, based on which participants are classified in categories of obesity.

^b^Different method of estimating obesity, the predictive power of which is compared to reference model/reference method.

^c^Number of participants.

^d^Number of participants that are positive with regard to respective outcome.

^e^Number of participants that are negative with regard to respective outcome.

^f^Percentage improvement (+) or deterioration (−) in predictive power of new model compared to reference model. Categories of obesity/FM% as independent variable.

^g^Net reclassification of cases + net reclassification of noncases. A positive number denotes increased predictive power for the new model.

^h^Likelihood of net reclassification index to be 0, that is, the new model showing no improvement/deterioration over reference model.

^i^Net reclassification of cases = percentage of cases reclassified by the new model into a higher risk category − percentage of cases reclassified by the new model into a lower risk category

^j^Net reclassification of non-cases = percentage of non-cases reclassified by the new model into a lower risk category − percentage of non-cases reclassified by the new model into a higher risk category.

^k^Integrated discrimination improvement (+) or deterioration (−) of new model compared to reference model. Categories of obesity/FM% as independent variable in an age-adjusted model.

^l^Mean difference in predicted individual probabilities between cases and non-cases for two models. A positive number denotes increased predictive power for the new model.

^m^Likelihood of net reclassification index to be 0, that is, the new model showing no improvement/deterioration over reference model.

^n^Measures of obesity (BMI/FM%) as continuous variable in a logistic regression model predicting respective outcomes.

^o^Difference in area under curve of receiver operating characteristic compared to reference method.

^p^Probability of 0-hypothesis (no difference).

^q^Definitions of hypertension according to European Societies for Hypertension and Cardiology {Mancia, 2007 #2897}.

^r^Estimation of FM% with bioimpedance device InBody (720) (Biospace, Korea).

^s^Anthropometry-based estimate; arithmetic mean of FM% estimations according to prediction methods Deurenberg et al. [12], Gallagher et al. [15], and Larsson et al. [14].

^t^Triacylglycerols ≥ 1.7 mmol/L or HDL cholesterol ≤ 1.29 mmol/L in men or HDL ≤ 1.03 mmol/L in women.

**Table 5:** Prediction/discrimination of impaired fasting glucose and the metabolic syndrome with degree of obesity as defined by dual-energy X-ray absorptiometry (DXA) bioimpedance analysis (BIA), an anthropometry-based estimate of fat mass percentage (FM%-equation) and BMI.

										ROC analyses^n^			
Reference method/model^a^	New method/model^b^	*n* ^c^		Reclassification index, %^f^				IDI, %^k^		Men		Women	
		Cases^d^	Non-cases^e^	Net^g^	*P*_nri_^h^	Cases^i^	Non-cases^j^	*I*_integr._^l^	*P*_idi_^m^	Δ AUC^o^	*P* ^p^	Δ AUC	*P*
Impaired fasting glucose (≥5.6 mmol/L = 100 mg/dL)													
DXA	BIA InBody^q^	164	249	−6%	0.181	−7%	1%	−0.5%	0.506	−0.03	0.102	−0.01	0.394
	BMI	191	276	−2%	0.727	−4%	2%	0.3%	0.723	−0.04	0.286	−0.02	0.394
	Estimate^r^	191	276	−1%	0.771	−4%	2%	0.2%	0.809	−0.03	0.404	−0.01	0.597
BIA InBody	BMI	164	249	2%	0.752	1%	0%	0.2%	0.796	−0.01	0.888	−0.01	0.744
	Estimate	164	249	1%	0.769	1%	1%	−0.1%	0.882	0.00	0.890	0.00	0.981
BMI	Estimate	191	276	0%	1.000	0%	0%	−0.1%	0.733	0.01	0.514	0.01	0.386

Impaired fasting glucose (≥6.1 mmol/L = 110 mg/dL)													
DXA	BIA InBody	70	343	−1%	0.901	−4%	3%	3.5%	0.009	−0.01	0.584	0.01	0.462
	BMI	80	387	6%	0.394	3%	4%	3.2%	0.009	0.00	0.900	0.00	0.918
	Estimate	80	387	3%	0.616	0%	3%	2.6%	0.023	0.02	0.438	0.01	0.796
BIA InBody	BMI	70	343	7%	0.341	6%	1%	−0.7%	0.609	0.02	0.648	−0.02	0.504
	Estimate	70	343	2%	0.754	1%	1%	−1.5%	0.205	0.04	0.253	−0.01	0.799
BMI	Estimate	80	387	−3%	0.251	−3%	-1%	−0.6%	0.176	0.02	0.315	0.01	0.248

Metabolic syndrome (AHA/NHBLI)^s^													
DXA	BIA InBody	144	268	−4%	0.400	−6%	2%	−0.7%	0.691	−0.03	0.120	0.01	0.625
	BMI	165	301	4%	0.461	0%	4%	2.5%	0.257	−0.02	0.610	0.02	0.309
	Estimate	165	301	3%	0.519	−1%	4%	1.7%	0.407	−0.02	0.466	0.02	0.329
BIA InBody	BMI	144	268	3%	0.595	2%	1%	0.9%	0.662	0.01	0.697	0.01	0.429
	Estimate	144	268	4%	0.480	2%	1%	0.8%	0.681	0.01	0.812	0.01	0.409
BMI	Estimate	165	301	−1%	0.577	−1%	0%	−0.7%	0.252	−0.01	0.622	0.00	0.958

^a^Method of measurement, based on which participants are classified in categories of obesity.

^b^Different method of estimating obesity, the predictive power of which is compared to reference model/reference method.

^c^Number of participants.

^d^Number of participants that are positive with regard to respective outcome.

^e^Number of participants that are negative with regard to respective outcome.

^f^Percentage improvement (+) or deterioration (−) in predictive power of new model compared to reference model. Categories of obesity/FM% as independent variable.

^g^Net reclassification of cases + net reclassification of non-cases. A positive number denotes increased predictive power for the new model.

^h^Likelihood of net reclassification index to be 0, that is, the new model showing no improvement/deterioration over reference model.

^i^Net reclassification of cases = percentage of cases reclassified by the new model into a higher risk category − percentage of cases reclassified by the new model into a lower risk category.

^j^Net reclassification of non-cases = percentage of non-cases reclassified by the new model into a lower risk category − percentage of non-cases reclassified by the new model into a higher risk category.

^k^Integrated discrimination improvement (+) or deterioration (−) of new model compared to reference model. Categories of obesity/FM% as independent variable in an age-adjusted model.

^l^Mean difference in predicted individual probabilities between cases and non-cases for two models. A positive number denotes increased predictive power for the new model.

^m^Likelihood of net reclassification index to be 0, that is, the new model showing no improvement/deterioration over reference model.

^n^Measures of obesity (BMI/FM%) as continuous variable in a logistic regression model predicting respective outcomes.

^o^Difference in area under curve of receiver operating characteristic compared to reference method.

^p^Probability of 0-hypothesis (no difference).

^q^Estimation of FM% with bioimpedance device InBody (720) (Biospace, Korea).

^r^Anthropometry-based estimate; arithmetic mean of FM% estimations according to prediction methods Deurenberg et al. [12], Gallagher et al. [15], and Larsson et al. [14].

^s^Definition of metabolic syndrome suggested by the common task force from the IDF and the American Heart Association/National Heart, Lung and Blood Institute (AHA/NHBLI) [17].
